# On the equivalence of demagnetization tensors as discrete cell size approaches zero in three-dimensional space

**DOI:** 10.1063/5.0226603

**Published:** 2024-08-26

**Authors:** Hao Liang, Xinqiang Yan

**Affiliations:** 1 Vanderbilt University Institute of Imaging Science, Vanderbilt University Medical Center, Nashville, Tennessee 37232, USA; 2 Department of Radiology and Radiological Sciences, Vanderbilt University Medical Center, Nashville, Tennessee 37232, USA; 3 Department of Electrical and Computer Engineering, Vanderbilt University, Nashville, Tennessee 37232, USA

## Abstract

The calculation of the demagnetization field is crucial in various disciplines, including magnetic resonance imaging and micromagnetics. A standard method involves discretizing the spatial domain into finite difference cells and using demagnetization tensors to compute the field. Different demagnetization tensors can result in contributions from adjacent cells that do not approach zero, nor do their differences, even as the cell size decreases. This work demonstrates that in three-dimensional space, a specific set of magnetization tensors produces the same total demagnetization field as the Cauchy principal value when the cell size approaches zero. Additionally, we provide a lower bound for the convergence speed, validated through numerical experiments.

## I. INTRODUCTION

Calculation of magnetic fields is important in various fields, including micromagnetics and magnetic resonance imaging (MRI). In the field of micromagnetics, numerical simulations often solve the Landau–Lifshitz–Gilbert equation along with its associated partial differential equations.^1^ The primary variable is the magnetization vector field, M. Given the magnetization field, various fields such as exchange, anisotropy, Zeeman, and demagnetization fields are computed,^2^ which, in turn, influence magnetization. The most computationally demanding part is calculating the demagnetization field given the magnetization.

In MRI, the strong magnetic fields produced by scanners create varying magnetizations within different tissue types in the body. These magnetizations result in non-uniformly distributed demagnetization fields. The presence of such magnetic fields inhomogeneities is a major challenge in acquiring high-quality images, especially at higher field strengths where demagnetization fields are also of greater amplitude. Accurately computing these fields is essential for understanding how they vary across individuals with different body sizes, shapes, and anatomical structures.

In the above-mentioned disciplines, a common calculation involves determining the demagnetizing field given the magnetization. A common approach is to discretize the space into a regular grid of cubic cells. Within each cell, the magnetization and other fields are considered constant. To calculate the demagnetization field, an integral over the entire magnetic domain, in finite difference discretization, translates to a summation over all cells as follows:^3^

$$H{\left(r\right)}^{\text{e}xclude\;cell\;at\;r}=-\sum_{r^{\prime }\neq r}N(r^{\prime }-r)\cdot M(r^{\prime }),$$ (1)

where N is the demagnetization tensor. The cell at $r^{\prime }=r$ is excluded from the summation for convenient comparison among different methods. Due to the approximation of the discretization, there are field errors, especially in the case of rapid changes in magnetization. However, the impact of discretization (e.g., cell size) on field errors has not yet been studied based on a comprehensive theoretical analysis. This article aims to fill this gap and provides a comprehensive theoretical explanation of these errors.

Typically, in micromagnetics, the demagnetization tensor is defined based on the interaction energy between cells, assuming each cubic cell is uniformly magnetized.^4–6^ This approach is referred to as the *uniformly magnetized cube (UMC)* method, and the demagnetization tensor of this approach is denoted as $N_{\text{c}}$. Another approach treats each cell as a point dipole located at its center. This approach is used in both micromagnetics^7,8^ and MRI^9–11^ owing to its simplicity of calculation for the demagnetization tensor. This approach is referred to as the *dipole* method, and the demagnetization tensor of this approach is denoted as $N_{\text{d}}$. Besides the *UMC* and *dipole* methods, the cell at $r^{\prime }$ can be treated as a uniformly magnetized cube; however, the cell at r is treated as a point dipole.^12^ This method can benefit from averaging $r^{\prime }$ over a cell and keeping the calculation relatively simple. We refer to this method as the *uniformly magnetized cube-dipole (UMCD)* method and its demagnetization tensor as $N_{\text{c}d}$.

**FIG. 1.**
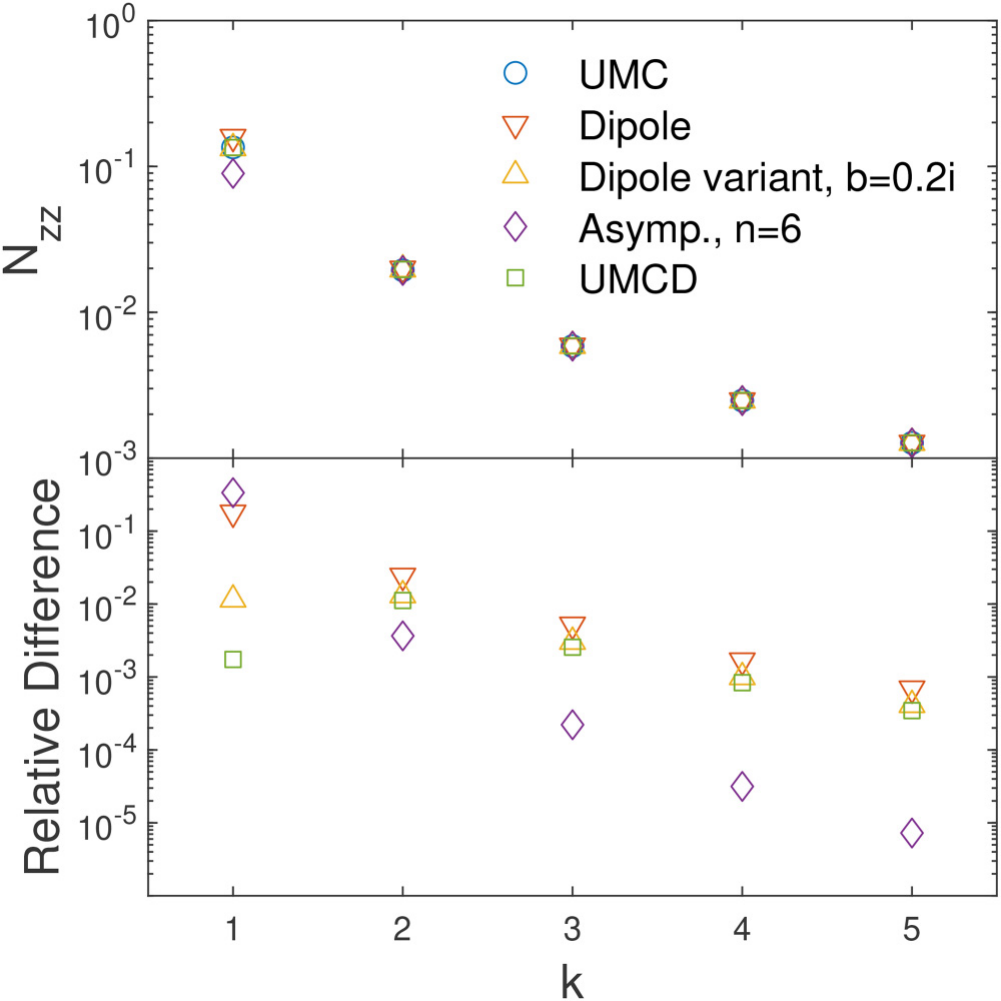
Upper panel: demagnetization tensor, $N_{zz}$, for the *UMC* method, *dipole* method, variant of *dipole* method with b=0.2ı, the asymptotic expansion for the *UMC* method up to n=6, i.e., $O\left({\left(h/r\right)}^{7}\right)$, and *UMCD* method. In the plots, $x-x^{\prime }=y-y^{\prime }=0$, and k is defined by $z^{\prime }-z=kh$. Lower panel: the relative differences between these methods and the *UMC* method. Specifically, the relative difference is defined as $\left|N_{zz}/{\left(N_{\text{c}}\right)}_{zz}-1\right|$ for demagnetization tensor N.

The equivalence of these methods is non-trivial. The demagnetization tensor is proportional to the volume of the small cell ( $h^{3}$) and also $-\nabla \nabla (1/|r|)∼(r^{2}-3z^{2})/r^{5}$, which is about $h^{-3}$ for cells adjacent to the cell where the field is being evaluated. This means that the demagnetization field contribution from *a single nearby cell* is *NOT* small but finite. This situation differs significantly from integration on a smooth function, where each piece is small, and the sum of small pieces is a finite value. In our situation, the contribution of a single cell adjacent to the cell at r does not approach zero, nor does the difference between different methods, even as the cell size approaches zero. For example, while $N_{\text{d}}$ is a good approximation of $N_{\text{c}}$ for $r^{\prime }$ far from r, this is not the case for cells adjacent to the cell at r. For example, the component ${\left(N_{\text{d}}\right)}_{zz}$ of the tensor $N_{\text{d}}$ is given by

$${\left(N_{\text{d}}\right)}_{zz}(r)=\frac{1}{4\pi }\frac{i^{2}+j^{2}-2k^{2}}{\rho^{5}},$$ (2)

where i=x/h, j=y/h, and k=z/h. It should be noted that M and H have the same dimension, making the demagnetization tensor unitless in the metric system. A single cell at i=j=0 and k=1 with magnetization of $M_{z}$ contributes $2M_{z}/\left(4\pi \right)$ to the demagnetization field $H_{z}$. Similarly, using other methods, this single cell can contribute finite but different values to the demagnetization field. These differences can exceed ten percent, independent of h,^13,14^ as illustrated in Fig. 1. Therefore, the conditions under which these methods yield the same result for the demagnetization field require investigation.

To our knowledge, the relationship between these methods is not sufficiently discussed. Numerous literature implicitly assumed that the *UMC*, *dipole*, and *UMCD* can yield the correct results, i.e., converge to the same result.^4–11^ However, other works hint that these methods cannot converge to the same result, due to significant discrepancy between the demagnetization tensors for individual cells.^13–16^ Additionally, some works treat the result of the *dipole* method as equivalent to that of the Cauchy principal value.^9–11^

One main goal of this article is to prove that all these methods consistently produce results at the limit of h approaching zero in three-dimensional space using cubic cells. The equivalence is not on a cell-by-cell basis but on the total field for sufficiently smooth magnetization.

This article is structured as follows: Sec. II reviews the demagnetization tensors, presents the proof of our statement, provides a lower bound on the convergence speed, and outlines the implementation using FFT. Section III demonstrates the validation of the statement through numerical experiments. Section IV discusses the extension and limitation of the current work. Section V offers concluding remarks.

## II. THEORETICAL ANALYSIS

### A. Cauchy principal value

The demagnetization field for open boundary conditions is given by^2,3^

H=−∇ψ(r), (3)

$$\psi (r)=-\frac{1}{4\pi }\int \frac{\nabla^{\prime }\cdot M(r^{\prime })}{\left|r-r^{\prime }\right|}d^{3}r^{\prime },$$ (4)

where ∇ is applied to the functions with respect to r, and $\nabla^{\prime }$ is applied to the functions with respect to $r^{\prime }$, ψ is the scalar potential. Although the function $1/|r-r^{\prime }|$ with respect to $r^{\prime }$ diverges at r, the divergence is slow, and the integration of Eq. (4) exists as an improper integral. After some mathematical processing, Eq. (3) can be formally written as

$$H(r)=-\frac{1}{4\pi }\int M(r^{\prime })\cdot \nabla \nabla^{\prime }\frac{1}{\left|r-r^{\prime }\right|}d^{3}r^{\prime }.$$ (5)

However, the term $\nabla \nabla^{\prime }\left(1/\left|r-r^{\prime }\right|\right)$ as a function of $r^{\prime }$ diverges rapidly at r, and the result of Eq. (5) depends on how the singularity is treated. One approach is to use the Cauchy principal value, which we will briefly review. We decompose $M(r^{\prime })$ into two functions, $u^{-S}(r^{\prime })M(r^{\prime })$ and $u^{S}(r^{\prime })M(r^{\prime })$. Here, $u^{S}=1$ inside a sufficiently small sphere S, and $u^{S}=0$ elsewhere; conversely, $u^{-S}=1$ outside the sphere and $u^{-S}=0$ elsewhere. This ensures $u^{S}(r)+u^{-S}(r)\equiv 1$. Using this notation, ∫ always means the integral over the whole space, Eqs. (3) and (4) can be written as

$$H=\frac{1}{4\pi }\nabla \left(\int \frac{\nabla^{\prime }\cdot u^{-S}(r^{\prime })M(r^{\prime })}{\left|r-r^{\prime }\right|}d^{3}r^{\prime }+\int \frac{\nabla^{\prime }\cdot u^{S}(r^{\prime })M(r^{\prime })}{\left|r-r^{\prime }\right|}d^{3}r^{\prime }\right).$$ (6)

The surface magnetic charge has been implicitly included in Eq. (6).

Since $u^{-S}=0$ inside of the sphere, we can avoid the singularity issue in evaluating the first part in Eq. (6). The first part in Eq. (6) can be simplified as follows using the method of integration by parts:

$$\frac{1}{4\pi }\nabla \int \left(\nabla^{\prime }\cdot \frac{u^{-S}(r^{\prime })M(r^{\prime })}{\left|r-r^{\prime }\right|}-u^{-S}(r^{\prime })M(r^{\prime })\cdot \nabla^{\prime }\frac{1}{\left|r-r^{\prime }\right|}d^{3}r^{\prime }\right).$$ (7)

The first term in (7) is simply zero by Gauss’s law, noting that $u^{-S}M$ inside the sphere and around infinity is zero, respectively. Thus, Eq. (7) becomes

$$\begin{array}{rl} & -\frac{1}{4\pi }\nabla \int u^{-S}(r^{\prime })M(r^{\prime })\cdot \nabla^{\prime }\frac{1}{\left|r^{\prime }-r\right|}d^{3}r^{\prime }\\ & \;=-\frac{1}{4\pi }\int_{-S}M(r^{\prime })\cdot \nabla \nabla^{\prime }\frac{1}{\left|r-r^{\prime }\right|}d^{3}r^{\prime }. \end{array}$$ (8)

We identify this as the Cauchy principal value, denoted as $H_{\text{\textbackslash{},p}}$. The integration of the second term of the integrands in (6) is a well-known problem, and the result^17^ is $-\frac{1}{3}M$. The macroscopic field H can be decomposed into two parts,

$$H=-\frac{1}{3}M+H_{\text{p}}.$$ (9)

$H_{p}$ is given by

$$H_{\text{p}}(r)=-\int_{-S}N_{\text{p}}(r-r^{\prime })\cdot M(r^{\prime })d^{3}r^{\prime },$$ (10)

where

$$\begin{array}{rl} N_{\text{p}}(r-r^{\prime }) & =\frac{1}{4\pi }\nabla \nabla^{\prime }\frac{1}{\left|r^{\prime }-r\right|}\\ & =-\frac{1}{4\pi }\frac{3(r^{\prime }-r)(r^{\prime }-r)-|r^{\prime }-r{|}^{2}}{|r^{\prime }-r{|}^{5}} \end{array}$$ (11)

is the so-called 3×3 demagnetization tensor. ${\left(N_{\text{\textbackslash{},p}}\right)}_{ij}$ represents the field component in direction i at position r, generated by the magnetization component in direction j of a volume element at position $r^{\prime }$, where i and j are labels for axes. The integration of Eq. (11) is operational because the integration domain excludes the singularity point of $N_{\text{\textbackslash{},p}}$. It is important to note that the Cauchy principal value at r is distinct from the macroscopic magnetization by −M/3. More precisely, it represents the field observed in a spherical cavity generated from the remaining parts of magnetization.

In MRI, the magnetic field with the so-called sphere Lorentz correction, denoted as $B^{\prime }$ is of interest,^18^ namely,

$$B^{\prime }=B-\frac{2}{3}\mu_{0}M=\mu_{0}\left(H+\frac{1}{3}M\right).$$ (12)

Thus, we have $B^{\prime }=\mu_{0}H_{\text{p}}$, which is already discussed in the literature.^19,20^

### B. *UMC* and *UMCD* methods

As mentioned above, the *UMC* refers to the approach where each cubic cell is uniformly magnetized (however, the magnetization is not necessarily equal for different cells). In this method, the field is given by

$$H_{\text{c}}(r)=-\sum_{i,j,k}^{r_{ijk}\neq r}N_{\text{c}}(r_{ijk}-r)\cdot M(r_{ijk}),$$ (13)

$$r_{ijk}=(ie_{x}+je_{y}+ke_{z})h,$$ (14)

where i, j, and k are indices for 3D grid, and h represents the cell size. To make the article more concise, we express it as follows:

$$H_{\text{c}}(r)=-\sum_{r^{\prime }\neq r}N_{\text{c}}(r^{\prime }-r)\cdot M(r^{\prime }).$$ (15)

The cell at $r^{\prime }=r$ is excluded from the summation for convenient comparison with other methods. The value of the demagnetization tensor is defined as the average demagnetization field in a cell centered at r, which is generated by another uniformly magnetized cubic cell (with a unit magnetization) centered at $r^{\prime }$. Specifically, the demagnetization tensor is given by

$$N_{\text{c}}(r)=\frac{1}{4\pi h^{3}}\int_{\text{c}ell\;0}d^{3}r^{\prime }\int_{\text{c}ell\;r}d^{3}r^{\prime \prime }\nabla^{\prime }\nabla^{\prime \prime }\frac{1}{\left|r^{\prime \prime }-r^{\prime }\right|}.$$ (16)

The demagnetization tensor $N_{\text{c}}$ can be calculated using various methods, including numerical integration,^4,21^ exact analytical formulas,^1,3,14,15^ or analytical formulas combined with asymptotic expansions.^16^ As an example, $N_{zz}$ is calculated and shown in Fig. 1.

For the *UMCD* method, the cell at $r^{\prime }$ is treated as a uniformly magnetized cube, but the cell at r is treated as a point dipole. This results in a demagnetization tensor of

$$N_{\text{c}d}(r)=\frac{1}{4\pi }\int_{\text{c}ell\;r}d^{3}r^{\prime \prime }{\left(\nabla^{\prime }\nabla^{\prime \prime }\frac{1}{\left|r^{\prime \prime }-r^{\prime }\right|}\right)|}_{r^{\prime }=0}.$$ (17)

The analytical expression of the demagnetization tensor for the *UMCD* method^12,15^ is simpler than that for the *UMC*. $N_{\text{zz}}$ as an example is shown in Fig. 1. Similar to the *UMC* method, the cell at $r^{\prime }=r$ is excluded from the summation for the *UMCD* method, for convenient comparison with other methods.

However, for both methods, by noticing that $\text{Tr}\left[\nabla \nabla^{\prime }\left(1/\left|r^{\prime }-r\right|\right)\right]=\nabla \cdot \left(\nabla^{\prime }\left(1/\left|r^{\prime }-r\right|\right)\right)=4\pi \delta (r^{\prime }-r)$ and considering the symmetries of x, y, and z axes, it is not difficult to demonstrate that demagnetization tensor for cell at $r^{\prime }=r$ is $(1/3)\delta_{ab}$, where $\delta \left(r-r^{\prime }\right)$ is the Dirac delta function, $\delta_{ab}$ Kronecker delta, Tr denotes the trace of a 3×3 tensor. Therefore, the demagnetization field generated by the cell itself is −(1/3)M. Thus,

$$H_{\text{c}}^{\text{\textbackslash{},}full}\equiv -\sum_{r^{\prime }}N_{\text{c}}(r-r^{\prime })\cdot M(r^{\prime })=H_{\text{c}}(r)-\frac{1}{3}M$$ (18)

and

$$H_{\text{c}d}^{\text{\textbackslash{},}full}\equiv -\sum_{r^{\prime }}N_{\text{c}d}(r-r^{\prime })\cdot M(r^{\prime })=H_{\text{c}d}(r)-\frac{1}{3}M.$$ (19)

### C. *Dipole* method

As mentioned above, the *dipole* method replaces a uniformly magnetized cubic cell with a single dipole at the cell’s center. The demagnetization field is expressed by

$$H_{\text{d}}(r)=-\sum_{r^{\prime }\neq r}N_{\text{d}}(r^{\prime }-r)\cdot M(r^{\prime }),$$ (20)

where $N_{\text{d}}$ represents the demagnetization tensor for the *dipole* method. This tensor quantifies the demagnetization field at r, generated by a dipole with a moment of $h^{3}$ at $r^{\prime }$, specifically,

$$N_{\text{d}}(r)=-\frac{3}{4\pi \rho^{5}}\left(\begin{array}{ccc} i^{2}-\rho^{2}/3 & ij & ik\\ ij & j^{2}-\rho^{2}/3 & jk\\ ik & jk & k^{2}-\rho^{2}/3 \end{array}\right),$$ (21)

$$\rho \equiv \sqrt{i^{2}+j^{2}+k^{2}},$$ (22)

$$r=(ie_{x}+je_{y}+ke_{z})h,$$ (23)

where i, j, and k are integral indices for 3D grids; $e_{x}$, $e_{y}$, and $e_{z}$ are the unit vectors along x, y, and z directions, respectively.

Note that Eq. (21) does not explicitly depend on h. Therefore, the contribution from *a single cell* adjacent to the cell at r does *NOT* approach zero as h→0. It is also noteworthy that since the interaction energy between two uniformly magnetized spheres equals that between two dipoles of the same moment,^3,22,23^ the method can also be referred to as the *uniformly magnetized sphere* method. $r^{\prime }=r$ must be excluded from the summation because the demagnetization tensor for the *dipole* method is singular at this point.

### D. Asymptotic expansions

The demagnetization tensors for both *UMC* and *UMCD* methods have an asymptotic expansion in the form of (see Appendix A)

$$\sum_{n}N^{\left(n\right)}(r)=\sum_{n=2,4,\ldots }\sum_{s,t}c_{n,s,t}{\left(\frac{x}{r}\right)}^{s}{\left(\frac{y}{r}\right)}^{t}{\left(\frac{z}{r}\right)}^{n-s-t}{\left(\frac{h}{r}\right)}^{n+1},$$ (24)

where $c_{n,s,t}$ are coefficients of mathematical constants. It can also be written in the form of

$$\sum_{n=2,4,\ldots }\sum_{s,t}c_{n,s,t}{\left(\frac{i}{\rho }\right)}^{s}{\left(\frac{\;j}{\rho }\right)}^{t}{\left(\frac{k}{\rho }\right)}^{n-s-t}{\left(\frac{1}{\rho }\right)}^{n+1},$$ (25)

where i, j, k, ρ are defined by x/h, y/h, z/h, and |r|/h, respectively. Again, h does not explicitly appear in the formula. Evaluating $c_{n,i,j}$ according to Appendix A for both *UMC* or *UMCD* methods, we find that: for n=2, this represents the dipole approximation; For n=4, these values are zero for cubic cells, which explains why the demagnetization tensor of *dipole* method converges to those of the *UMC* or *UMCD* fast for distant cells. For n=6, the asymptotic expansions for $N_{\text{c}}$, such as the components $N_{xx}^{\left(6\right)}$ and $N_{xy}^{\left(6\right)}$, are given as follows:

$$\begin{array}{r} N_{xx}^{\left(6\right)}=\frac{1}{4\pi }\cdot \frac{7}{16\rho^{13}}\left(2i^{6}-j^{6}-k^{6}-15i^{4}(j^{2}+k^{2})+15i^{2}(j^{4}+k^{4})\right) \end{array}$$ (26)

and

$$\begin{array}{r} N_{xy}^{\left(6\right)}=\frac{1}{4\pi }\cdot \frac{7}{16\rho^{13}}ij\left(7i^{4}-19i^{2}j^{2}+7j^{4}-13(i^{2}+j^{2})k^{2}+13k^{4}\right). \end{array}$$ (27)

For the *UMCD* method, the correction of n=6 to the demagnetization tensor is only half of that for the *UMC* method. The asymptotic expansion of the magnetization tensor for the *UMC* is shown in Fig. 1. The figure shows that the asymptotic expansions for the *UMC* method up to n=6 converge much faster than the dipole approximation for distant cells. However, higher-order corrections increase the error for the cell adjacent to the cell where the field is being evaluated.

### E. Near and far field decomposition

We use an analysis method similar to that described in the literature^17^ to split the demagnetization field into near and far fields. The near magnetic field originates from a cubic volume V of size L×L×L, while the far field encompasses contributions outside this cubic volume. We can always choose L to be macroscopically small but significantly larger than h, ensuring that V contains exactly 2N+1 cubic cells, satisfying

$$h\ll 1,\;L\ll 1,\;\text{a}nd\;\frac{L}{h}=2N+1\gg 1.$$ (28)

We will prove that these methods result in the same field under the limit

$$h\to 0,\;L\to 0,\;\text{a}nd\;\frac{L}{h}\to +\infty .$$ (29)

It is intuitive that these methods yield the same far field, given that L/h→∞ under the limit defined in Eq. (29). Therefore, it is only necessary to prove these methods produce the same near field under the limit (29).

### F. Near field for uniform magnetization

First, we prove that the near part of the demagnetization field produced by all these methods equals zero in the case of uniform magnetization, i.e., the magnetization is uniform in the near cells. We utilize a similar treatment as in literature.^17^ For the Cauchy principal value method, the near field can be expressed as

$$H_{\text{\textbackslash{},}p}^{V}(r)=-\int_{V-S}N_{\text{\textbackslash{},}p}(r-r^{\prime })\cdot M_{0}(r^{\prime })d^{3}r^{\prime }.$$ (30)

The superscript V indicates that only the contribution from the cubic volume V is counted. We can directly verify that ${\left(N_{\text{\textbackslash{},p}}\right)}_{xx}(r-r^{\prime })+{\left(N_{\text{\textbackslash{},p}}\right)}_{yy}(r-r^{\prime })+{\left(N_{\text{\textbackslash{},p}}\right)}_{zz}(r-r^{\prime })=\delta (r^{\prime }-r)$ according to (11), thus

$$\int_{V-S}\left({\left(N_{\text{\textbackslash{},}p}\right)}_{xx}+{\left(N_{\text{\textbackslash{},}p}\right)}_{yy}+{\left(N_{\text{\textbackslash{},}p}\right)}_{zz}\right)d^{3}r^{\prime }=0.$$ (31)

Since r is exactly at the center of V, there is the symmetry between x, y, and z axes. Thus, the integral of the three terms in (31) are equal and must be equal to zero, namely,

$$\int_{V-S}{\left(N_{\text{\textbackslash{},}p}\right)}_{xx}d^{3}r^{\prime }=\int_{V-S}{\left(N_{\text{\textbackslash{},}p}\right)}_{yy}d^{3}r^{\prime }=\int_{V-S}{\left(N_{\text{\textbackslash{},}p}\right)}_{zz}d^{3}r^{\prime }=0.$$ (32)

For the *dipole* method, we need to evaluate the magnetic field from dipoles inside the cube, according to (20), namely,

$$H_{\text{d}}^{V}(r)=-\sum_{\begin{array}{c} r^{\prime }\in V\\ r^{\prime }\neq r \end{array}}N_{\text{d}}(r-r^{\prime })\cdot M(r^{\prime }).$$ (33)

We can directly examine ${\left(N_{\text{d}}\right)}_{xx}+{\left(N_{\text{d}}\right)}_{yy}+{\left(N_{\text{d}}\right)}_{zz}=0$ according to (21), thus,

$$\sum_{\begin{array}{c} r^{\prime }\in V\\ r^{\prime }\neq r \end{array}}^{N}({\left(N_{\text{d}}\right)}_{xx}+{\left(N_{\text{d}}\right)}_{yy}+{\left(N_{\text{d}}\right)}_{zz})=0.$$ (34)

Since the symmetry between x, y, and z axes, the summations of each of three terms in (34) are equal to zero, as follows:

$$\sum_{\begin{array}{c} r^{\prime }\in V\\ r^{\prime }\neq r \end{array}}{\left(N_{\text{d}}\right)}_{xx}=\sum_{\begin{array}{c} r^{\prime }\in V\\ r^{\prime }\neq r \end{array}}{\left(N_{\text{d}}\right)}_{yy}=\sum_{\begin{array}{c} r^{\prime }\in V\\ r^{\prime }\neq r \end{array}}{\left(N_{\text{d}}\right)}_{zz}=0.$$ (35)

Due to the parity (mirror) symmetry, the off diagonal elements of the demagnetization tensor are also zero

$$\int_{V-S}{\left(N_{\text{\textbackslash{},}p}\right)}_{xy}d^{3}r^{\prime }=\int_{V-S}{\left(N_{\text{\textbackslash{},}p}\right)}_{yz}d^{3}r^{\prime }=\int_{V-S}{\left(N_{\text{\textbackslash{},}p}\right)}_{xz}d^{3}r^{\prime }=0,$$ (36)

$$\sum_{\begin{array}{c} r^{\prime }\in V\\ r^{\prime }\neq r \end{array}}{\left(N_{\text{d}}\right)}_{xy}=\sum_{\begin{array}{c} r^{\prime }\in V\\ r^{\prime }\neq r \end{array}}{\left(N_{\text{d}}\right)}_{yz}=\sum_{\begin{array}{c} r^{\prime }\in V\\ r^{\prime }\neq r \end{array}}{\left(N_{\text{d}}\right)}_{xz}=0.$$ (37)

For the *UMC*, *UMCD*, and their asymptotic expansion methods, the symmetries also exist, allowing similar equations to Eqs. (35) and (37) to hold. Note that the *dipole* method can be seen as the leading order of *UMC* and *UMCD*’s asymptotic expansions. In summary, we have proven that, for all these methods, the near part of the demagnetization field is exactly zero, namely,

$$H_{\text{\textbackslash{},}p}^{V}(r)=H_{\text{c}}^{V}(r)=H_{\text{c}d}^{V}(r)=H_{\text{d}}^{V}(r)=0.$$ (38)

Modifications to the demagnetization tensors, while preserving Eq. (34) and symmetries of x, y, and z axes, can also result in (35). This guides us in creating variation of the *dipole* method. An example is replacing $\rho =\sqrt{i^{2}+j^{2}+k^{2}}$ in the denominator in Eq. (21) with $\rho^{\prime }=\sqrt{i^{2}+j^{2}+k^{2}+b}$, namely,

$$N_{\text{d}}(r)=-ℜ\frac{3}{4\pi \rho^{\prime 5}}\left(\begin{array}{ccc} i^{2}-\rho^{2}/3 & jk & ik\\ jk & j^{2}-\rho^{2}/3 & jk\\ ik & jk & k^{2}-\rho^{2}/3 \end{array}\right),$$ (39)

$$\rho =\sqrt{i^{2}+j^{2}+k^{2}},\;\rho^{\prime }=\sqrt{i^{2}+j^{2}+k^{2}+b},$$ (40)

where b can be positive real number or imaginary number, ℜ⋆ denote the real part of ⋆. The additional term b softens the demagnetization tensor, possibly leading to a smoother result where M changes rapidly.

### G. Near field for nonuniform magnetization

In the previous subsection, it has been established that the near demagnetization field is exactly zero for uniform magnetization. Then, we prove that the near demagnetization field at r approaches zero as L→0, provided that the magnetization is Hölder continuous at r.

By subtracting a constant magnetization, $M_{0}=M(r)$, from $M(r^{\prime })$, the result remains unaffected. Consequently, with this subtraction, ${\left(M(r^{\prime })-M_{0})\right|}_{r^{\prime }=r}\;=0$, allowing us to potentially address the challenges arising from the discrepancy of the demagnetization tensor around r.

For the principal integral method, we have

$$H_{\text{\textbackslash{},}p}^{V}(r)=-\int_{V-S}N_{\text{\textbackslash{},}p}(r-r^{\prime })\cdot (M(r^{\prime })-M_{0})d^{3}r^{\prime }.$$ (41)

We assume $M(r^{\prime })$ hold Hölder condition at r, namely,

$$|M(r^{\prime })-M_{0}|<K|r^{\prime }-r{|}^{\alpha },$$ (42)

where K is a constant independent of $r^{\prime }$ and α>0.

First, we can estimate the magnitude of the demagnetization field for the principal integral method. Since $N_{\text{\textbackslash{},p}}$ diverges as $r^{-3}$ as r→0, the integral in spherical coordinates is bounded by

$$H_{\text{\textbackslash{},}p}^{V}∼\int_{V-S}Kr^{\alpha }(1/r^{3})r^{2}drd\theta d\phi ∼KL^{\alpha }.$$ (43)

In the estimation, we neglect constants on order of one. Equation (43) approaches zero as L→0. Then, we turn to the *dipole* method. Similarly, we can estimate the magnitude of the demagnetization field of the *dipole* method as

$$\begin{array}{rl} H_{\text{d}}^{V}(r) & ∼\sum_{i,j,k}K{\left(\rho h\right)}^{\alpha }(1/\rho^{3})\\ & ∼\sum_{I=1}^{N}\sum_{\max(|i|,|j|,|k|)=I}K{\left(Ih\right)}^{\alpha }(1/I^{3})\\ & ∼\sum_{I=1}^{N}K{\left(Ih\right)}^{\alpha }I^{2}/I^{3}\\ & ∼\sum_{I=1}^{N}Kh^{\alpha }I^{\alpha -1}\\ & ∼Kh^{\alpha }(N^{\alpha }-1)\\ & ∼KL^{\alpha }. \end{array}$$ (44)

Thus, $H_{\text{d}}^{V}(r)\to 0$ at limit of L→0.

The differences between the *dipole* method and other methods are bounded by higher-order terms in the asymptotic expansions, possibly multiplied by a factor of order one. For these higher-order corrections, we replace $\rho^{-3}$ with $\rho^{-(1+n)}$ in Eq. (44), where n≥4. After summing over all cells in V, the higher-order correction is on the order of $Kh^{\alpha }\times \text{max}\{1,N^{\alpha -(n-2)}\}$, where N∼L/h. Thus, the higher-order correction to the near field is smaller than or on the same order as that for the *dipole* method.

In summary, provided $M(r^{\prime })$ holds Hölder condition at r, all these methods result in a zero near demagnetization field as L→0, specifically

$$\lim_{L\to 0}H_{\text{d}}^{V}(r)=\lim_{L\to 0}H_{\text{c}}^{V}(r)=\lim_{L\to 0}H_{\text{c}d}^{V}(r)=\lim_{L\to 0}H_{\text{\textbackslash{},}p}^{V}(r)=0.$$ (45)

Note that to prove the demagnetization field is zero for a sufficiently small cubic volume centered at r, Eq. (42) is sufficient. It does not necessarily require Hölder continuity to hold everywhere in V.

### H. Lower bound on the convergence speed

We have proven that the near field is zero as L→0, assuming Hölder condition holds at r. All methods intuitively yield the same result for the far field; thus, a detailed analysis is not provided. Now, we give a lower bound on the convergence speed with the specific condition for magnetization, i.e., the Hölder condition holds at every cell. All these methods, including the exact Cauchy principal value method, yield values on the order of $KL^{\alpha }$ for the near fields, excluding $r^{\prime }=r$ from summation. Consequently, the error in the near field is of the order $KL^{\alpha }$. For the analysis of the far field, we begin with the *UMCD* method, in which each cell has an error on the order of $Kh^{\alpha }/\rho^{3}$. The total error for the far field is bounded by the order of

$$\sum_{i,j,k\notin V}\frac{Kh^{\alpha }}{\rho^{3}}∼\sum_{I\notin V}\frac{Kh^{\alpha }I^{2}}{I^{3}}∼Kh^{\alpha }\log(L^{\text{m}ax}/L),$$ (46)

where $L^{\text{max}}$ is the maximum material length. Taking L∼h, the total error of the near and far field is on the order of

$$Kh^{\alpha }\log(L^{\text{m}ax}/h).$$ (47)

For the other methods, the error has two parts: first, the error of the *UMCD* method, and second, the differences between these methods. For the far field, the difference between these methods is bounded by the asymptotic expansion of n=6, i.e., $|\max M|/\rho^{7}$ for a cell. After summing over all cells, the total error is bounded by the order of

$$|\max M|{\left(h/L\right)}^{4}.$$ (48)

The total error for the near field and the far field part of Eq. (48) is minimized to the order of

$$h^{4\alpha /(4+\alpha )},$$ (49)

for $L∼h^{4/(4+\alpha )}$. For $L∼h^{4/(4+\alpha )}$, the error of the far field for the *UMCD* method as in Eq. (46) is on the order of $Kh^{\alpha }\log(L^{\text{max}}/h)$, which is smaller than the error described in Eq. (49) and can be neglected. Given that this analysis is conservative and the errors are over-estimated, the actual convergence speed may exceed these estimates.

### I. Implementation using FFT

Noticing the translation symmetry property of the demagnetization tensor, the summation of the demagnetization tensor weighted by magnetization is a convolution operation. According to the cyclic convolution theorem, cyclic convolution can be performed using fast Fourier transform (FFT) and inverse FFT (IFFT) operations. To avoid the side effects of cyclic convolution, zero padding on the original array M is needed. The detailed steps are as follows: (1) Evaluate the array N of dimension $2N_{x}-1\times 2N_{y}-1\times 2N_{z}-1$ representing the demagnetization function, assuming r is at the center of the array. Then, circularly shift the center to the frontmost position. (2) Extend the dimension of M from $N_{x}\times N_{y}\times N_{z}$ to $\left(2N_{x}-1)\times (2N_{y}-1)\times (2N_{z}-1\right)$ by padding zeros on the end side. (3) Apply the cyclic convolution to M and N according to the cyclic convolution theorem,^24^

M⊗G=IFFT(FFT(M)×FFT(N)), (50)

where ⊗ is the cyclic convolution operation, × is the element-wise product operation. (4) Finally, clip the $N_{x}\times N_{y}\times N_{z}$ elements of M⊗G at the front. Except for rounding errors, the result would be identical to the direct summation. However, the time complexity is reduced from $O(N_{x}^{2}N_{y}^{2}N_{z}^{2})$ to $O(N_{x}N_{y}N_{z}\log(N_{x}N_{y}N_{z}))$ using FFT. Note the FFT(N) is independent of M, thus it can be reused for different Ms provided the dimension of the problem is unchanged.

## III. NUMERICAL VALIDATION

**FIG. 2.**
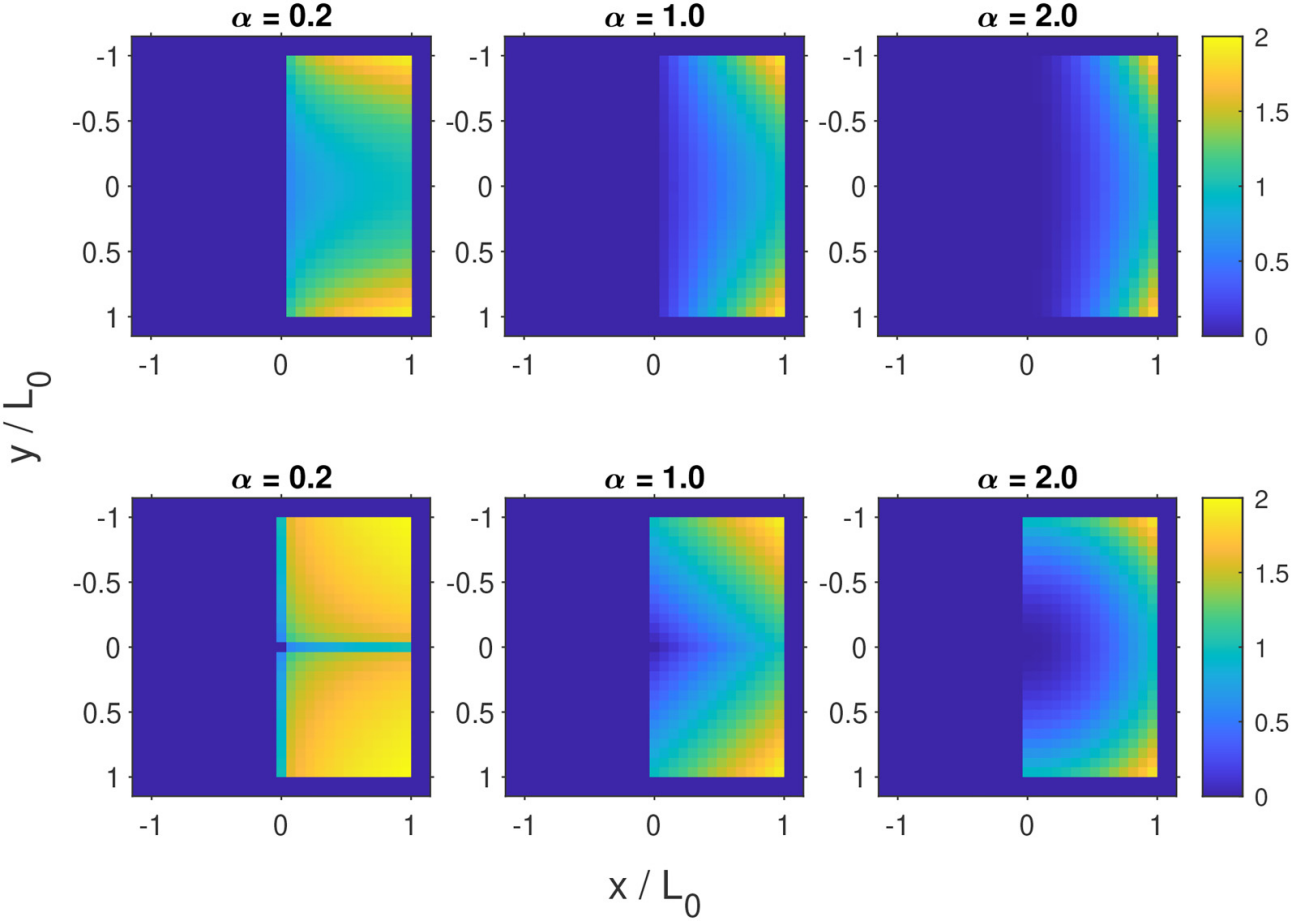
Upper panel: magnetization for z=0 described by Eq. (51) for different α’s. Lower panel: magnetization for z=0 described by Eq. (52) for different α’s.

To validate the conclusion that the three methods agree under certain conditions, we construct two problems. Problem I with magnetization

$$M=e_{z}M_{0}\left\{ \begin{array}{cc} |\eta_{x}{|}^{\alpha }(1+\eta_{y}^{2}+\eta_{z}^{2}) & \text{\textbackslash{},}for\;0\leq \eta_{x}\leq 1,|\eta_{y}|\leq 1,\text{and}|\eta_{z}|\leq 1,\\ 0\; & \;\text{o}therwise,\; \end{array}\right.$$ (51)

and problem II with magnetization

$$M=e_{z}M_{0}\left\{ \begin{array}{cc} |\eta_{x}{|}^{\alpha }+|\eta_{y}{|}^{\alpha }+|\eta_{z}{|}^{\alpha } & \text{\textbackslash{},}for\;0\leq \eta_{x}\leq 1,|\eta_{y}|\leq 1,\text{a}nd|\eta_{z}|\leq 1,\\ 0\; & \;\text{o}therwise,\; \end{array}\right.$$ (52)

where α>0, $\eta_{x}=x/L_{0}$, $\eta_{y}=y/L_{0}$, $\eta_{z}=z/L_{0}$, $L_{0}$ is the length of the magnet, $M_{0}$ is a constant with a unit of magnetization. The magnetizations are shown in Fig. 2. For problem I, the magnetization is discontinuous at the boundaries of the magnet at $x=L_{0}$, $z=\pm L_{0}$, and $y=\pm L_{0}$. While magnetization is continuous at the boundary of the magnet at x=0, its derivative is not for 0<α≤1. For problem I, at the center of each cell, r, we have $|M(r^{\prime })-M(r)|\leq 3M_{0}|r^{\prime }-r{|}^{\alpha }/L_{0}^{\alpha }$, thus it satisfies the Hölder continuous condition. For problem II, magnetization is discontinuous at the boundaries except for the point x=y=z=0. At x=y=z=0, magnetization is continuous; however, its derivative is not for 0<α≤1. The Hölder condition still holds at r=0.

For problem I, the exact analytical result can be expressed using special functions, as shown in Appendix B, with the aid of symbolic processing software.^25^ Thus, the result can be effectively calculated to arbitrary precision. We calculate the result to at least 20 digits, although the precision is truncated to about 16 digits when converting from a multi-precision representation to a double-precision floating-point format. For problem II, the exact result is zero for symmetry reasons.

On the numerical side, we employ several methods: the *UMC* method, the *dipole* method, and a variant of the *dipole* method described in Eq. (39) with b=0.2ı (where ı represents the imaginary unit), the asymptotic expansions for the *UMC* and *UMCD* methods. In the asymptotic expansion methods, the asymptotic expansions are used for both far and near cells. The coordinate system is set such that the origin is at the center of a cell. The cell size is adjusted so that at $x=L_{0}$, $y=\pm L_{0}$, and $z=\pm L_{0}$, the boundaries of the magnet align with the boundaries of the cells. At x=0, the cells’ centers are on the magnet’s boundary. Thus, we minimize the error fluctuations arising from the discretization of the magnet boundary. Computations are performed using double-precision floating-point arithmetic. The demagnetization tensors for the *UMC* and *UMCD* methods are calculated using exact formulas for cells at short distances and asymptotic expansions for cells at long distances. These asymptotic expansions include terms up to $O\left({\left(h/r\right)}^{7}\right)$. The demagnetization tensor has a maximum error on the order of $10^{-12}$ around the crossover between analytical formulas and asymptotic expansions. The numerical errors observed in this experiment, which are larger than $10^{-7}$, significantly exceed those of double-precision floating-point arithmetic ( $10^{-16}$) and the error of the demagnetization tensors. Therefore, all errors are attributed to discretization errors.

**FIG. 3.**
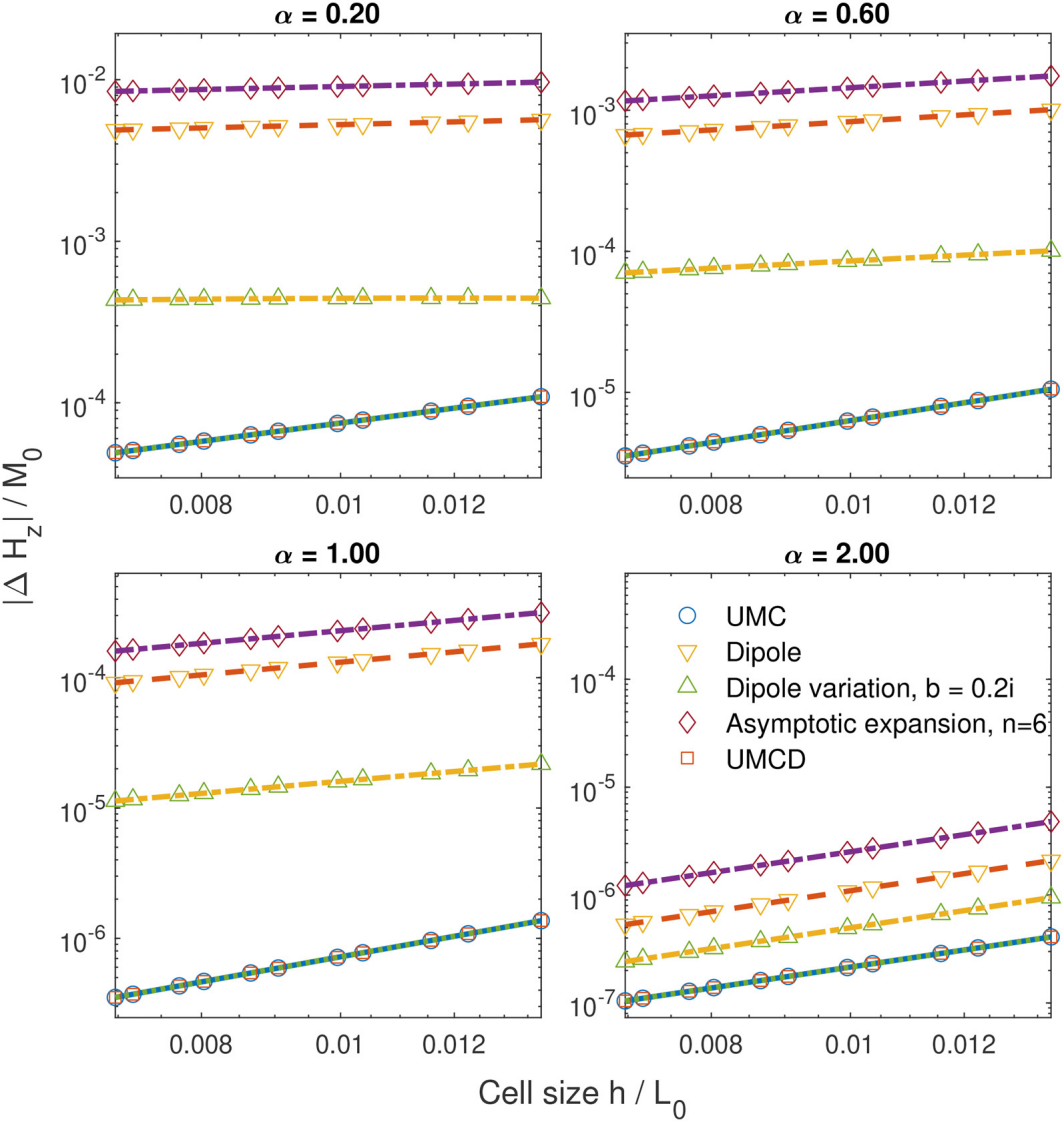
Error in the demagnetization field of numerical calculations at x=y=z=0 as a function of cell size using magnetization for problem I and for different values of α.

**FIG. 4.**
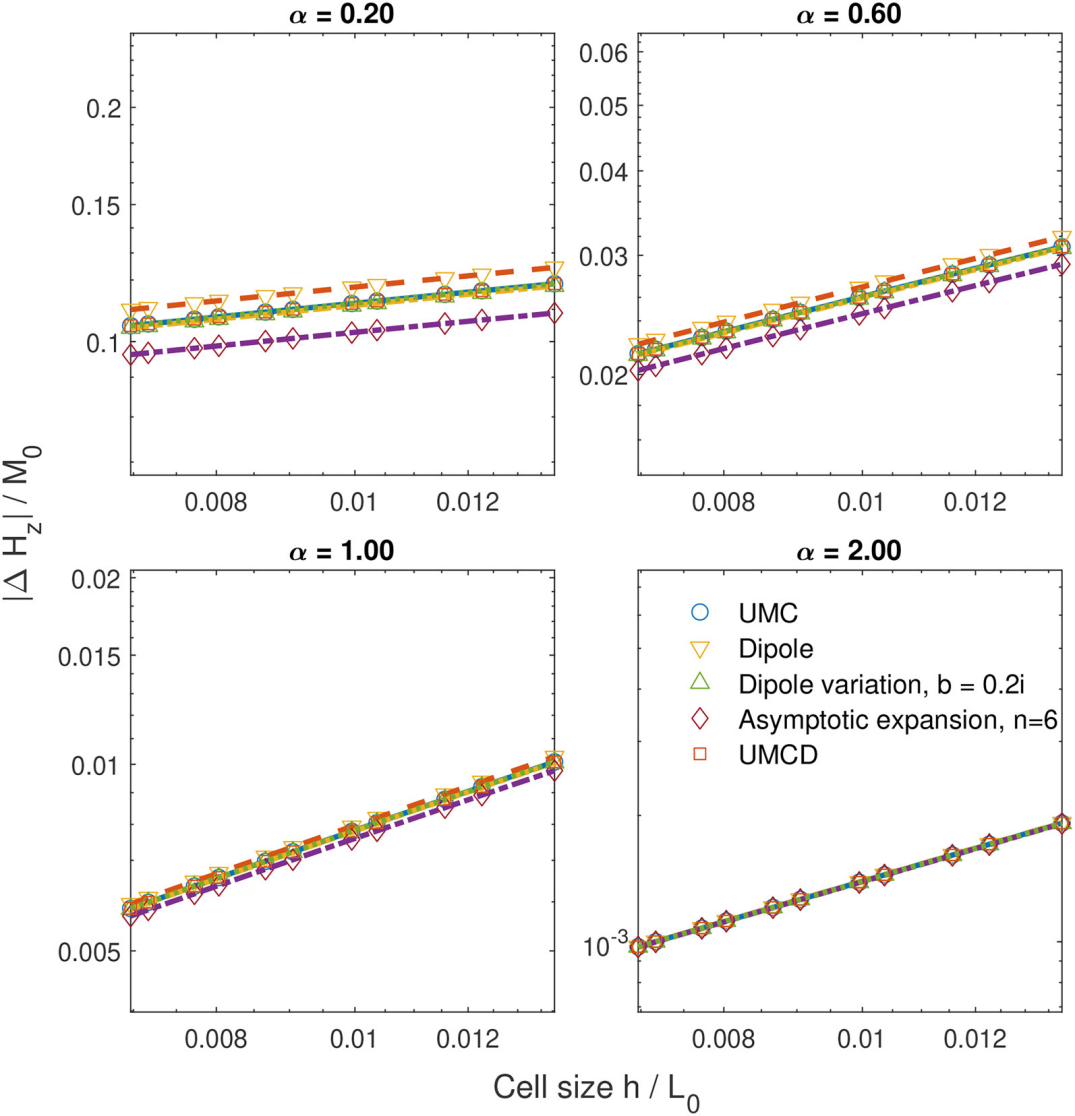
Error in the demagnetization field of numerical calculations at x=y=z=0 as a function of cell size using magnetization for problem II and for different values of α.

**FIG. 5.**
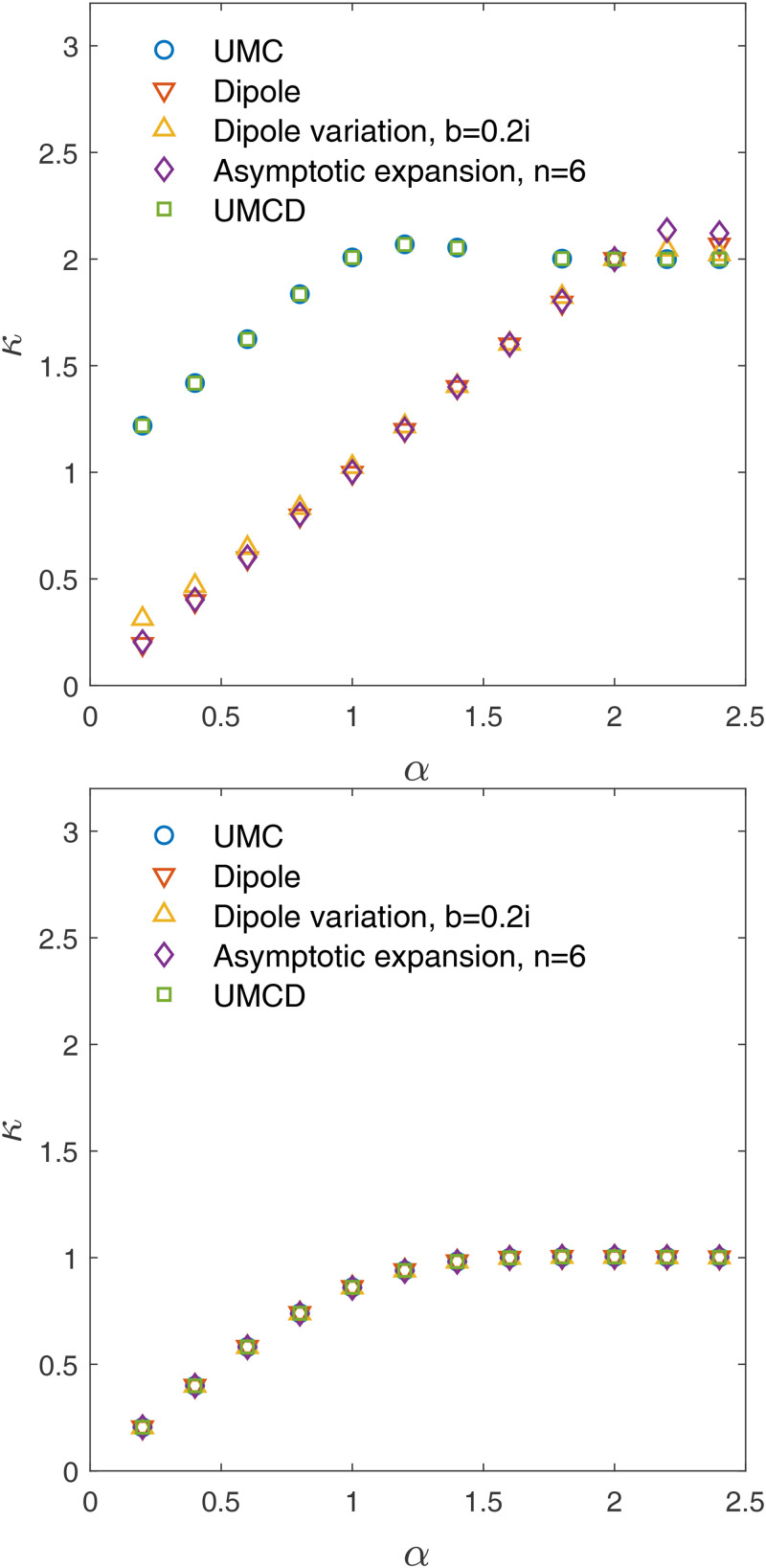
The converge speed as a function of α for problem I (upper panel) and problem II (lower panel).

The numerical errors of $H_{z}$ for α of 0.2, 0.6, 1, and 2 at r=0 are illustrated in Figs. 3 and 4. The numerical errors of $H_{z}$ are modeled by $c\cdot {\left(h/L_{0}\right)}^{\kappa +d{\left(\log(h/L_{0})\right)}^{-2}}$. The parameter κ determines the convergence speed as h→0. κ as a function of α are shown in Fig. 5. We observe that κ>0 for α>0. Thus, we verify that these methods converge to the same value for α>0, which is consistent with the prediction of our analysis. All convergence speeds slow down as α→0 where magnetization becomes more non-smooth. In problem I, the convergence speeds for the *UMC* method *UMCD* methods are much faster and have almost identical convergence speeds. These two methods are significantly better than the other methods. This can be explained as follows: for the magnetization relevant to the term $\eta_{x}^{\alpha }e_{z}$, the volume magnetic charge is zero and only surface magnetic charges appear on $z=\pm L_{0}$. For these two methods, the error can be attributed to the discretization error for the magnetic surface charge at the magnet surface of $z=\pm L_{0}$. This surface is far from where the field is under evaluation; thus, the error is suppressed. For the magnetization relevant to $\eta_{x}^{\alpha }(\eta_{y}^{2}+\eta_{z}^{2})e_{z}$, the discretization error from the near field is highly suppressed by the term $\eta_{y}^{2}+\eta_{z}^{2}$. Thus, these two methods show an advantage over the other methods. We also note that the variation of the *dipole* method shows a better convergence speed than the *dipole* method. The asymptotic expansion does not have an advantage compared to other method. This is as expected since the asymptotic expansions are not good for near cells. The convergence speed is faster than our conservative estimate; thus, our results are not violated. For α≥2, the κ value is close to 2 for all methods, consistent with other studies.^26,27^ In problem II, all these methods exhibit similarly slow convergence speeds. This issue can be attributed to the discontinuity at x=0, which worsens the results from all methods. Additionally, as α approaches zero, κ for all methods approaches zero, indicating non-convergence. This confirms the assertions of this work.

## IV. DISCUSSIONS

One might use a rectangular prism instead of a simple cube. For the uniformly magnetized prism method, the macroscopic demagnetization field can still be correctly calculated.^26^ However, in the *dipole* method, using different cell sizes along different directions will disrupt Eqs. (34) and (35), leading to discrepancies between the two methods. They represent different physics in this case. It is worth noting again that this discrepancy cannot be eliminated as cell size approaches zero.

For sufficiently smooth magnetization, the convergence speed estimated by Eqs. (47) and (49) is not faster than h. However, numerical experiments indicate a convergence speed of $h^{2}$ for sufficiently smooth magnetization. Therefore, the lower bound of the convergence speed is much worse than that observed in numerical experiments. To achieve a better lower bound estimate for the convergence speed, one may need methods beyond the simple near and far field splitting, and cell-by-cell analysis. Given the potential for error cancelation among cells, it is crucial to carefully analyze the correlations between errors of different cells.

The section of our proof that demonstrates the near field is zero for uniform magnetization closely resembles the approach described in the literature.^17^ However, it serves a different purpose: while the literature^17^ aims to establish the relationship between molecular polarizability and electric susceptibility, our goal is to prove that different magnetization tensors yield consistent results for both non-uniform and uniform magnetization.

The conclusion does not readily extend to two-dimensional materials in three-dimensional space. The magnetization of a two-dimensional thin material that is one cell thick cannot be considered a three-dimensional Hölder continuous function. Specifically, the symmetries between x, y, and z axes are broken for two-dimensional materials.

## V. CONCLUSION

Due to the discretization in numerical computation, the target object is typically divided into small cubic cells. The magnetic field at the target location is the sum of contributions from sources beyond the target cell. We proved that the *UMC*, *UMCD*, and *dipole* methods, their asymptotic expansions, and the Cauchy principal value method yield consistent results in three-dimensional space as the cell size approaches zero, namely,

$$\lim_{h\to 0}H_{\text{d}}(r)=\lim_{h\to 0}H_{\text{c}}(r)=\lim_{h\to 0}H_{\text{c}d}(r)=H_{\text{p}}.$$ (53)

If the self-contribution of the cell to the demagnetization field is included in both the *UMC* and the *UMCD* methods, then

$$\begin{array}{rl} \lim_{h\to 0}H_{\text{d}}(r)-\frac{1}{3}M & =\lim_{h\to 0}H_{\text{c}}^{\text{\textbackslash{},}full}(r)\\ & =\lim_{h\to 0}H_{\text{c}d}^{\text{\textbackslash{},}full}(r)=H_{\text{\textbackslash{},}p}-\frac{1}{3}M=H. \end{array}$$ (54)

The agreement among these demagnetization tensors is significant. In the calculation of the demagnetization tensor for the *UMC* method, both numerical integrals and lengthy exact analytical formulas introduce complexity in implementation. Additionally, exact analytical formulas can suffer from numerical stability issues, such as catastrophic cancelation.^16,21^ In contrast, the implementation of the demagnetization tensor in the *dipole* method is considerably simpler. For numerical calculations, the consistency among these methods allows us to employ the *dipole* method in situations where convergence speed is not a critical factor.

A comprehensive theoretical analysis is conducted to study the effect of discretization on field errors, offering valuable insights into the relationship between error amplitudes and cell size, addressing the scientific question. The theoretical analysis is presented in detail. The approach used is rigorous, comparing numerical solutions to analytical solutions to determine the errors. The findings in this manuscript are valuable to the micromagnetics and MRI communities, providing a better understanding of different methods for calculating demagnetization fields and how to use those methods more accurately.

We acknowledge that, while the conclusions are applicable to three-dimensional materials, further investigation is required for two-dimensional materials in three-dimensional space.

## Data Availability

The data that support the findings of this study are available from the corresponding author upon reasonable request.

## APPENDIX A: ASYMPTOTIC EXPANSIONS FOR THE *UMCD* AND *UMC* METHODS

We start from the *UMCD* method. Using $\nabla =-\nabla^{\prime }$ and substituting the following expansion:

$$\frac{1}{\left|r+r^{\prime }\right|}=\sum_{m=0}a_{n}\frac{{\left(2r\cdot r^{\prime }+|r^{\prime }{|}^{2}\right)}^{m}}{|r{|}^{2m+1}},$$ (A1)

into Eq. (17), the demagnetization tensor element ${\left(N_{\text{c}d}(r)\right)}_{ij}$ can be expressed in Cartesian coordinates as

$$\begin{array}{rl} -\frac{1}{4\pi } & \sum_{n=0}a_{m}\int_{-h/2}^{h/2}\int_{-h/2}^{h/2}\int_{-h/2}^{h/2}\text{d}x^{\prime }\text{d}y^{\prime }\text{d}z^{\prime }\\ & \times \left(\frac{\partial }{\partial x_{i}^{\prime }}\frac{\partial }{\partial x_{j}^{\prime }}\right)\frac{{\left(2(x^{\prime }x+y^{\prime }y+z^{\prime }z)+x^{\prime 2}+y^{\prime 2}+z^{\prime 2}\right)}^{m}}{r^{2m+1}}, \end{array}$$ (A2)

where $x_{1}^{\prime }=x^{\prime }$, $x_{2}^{\prime }=y^{\prime }$, and $x_{3}^{\prime }=z^{\prime }$. By substituting expansion of ${\left(2(x^{\prime }x+y^{\prime }y+z^{\prime }z)+x^{\prime 2}+y^{\prime 2}+z^{\prime 2}\right)}^{m}$ as

$$\sum_{\;p=0}^{m}b_{m,p}{(x^{\prime 2}+y^{\prime 2}+z^{\prime 2})}^{p}{(x^{\prime }x+y^{\prime }y+z^{\prime }z)}^{m-p},$$ (A3)

into (A2), we obtain a polynomial in h, x, y, z, and 1/r expressed as

$$\sum_{m}\sum_{\;p=0}^{m}d_{m,p,s,t}\frac{h^{2p+m-p+3-2}x^{m-p-s-t}y^{s}z^{t}}{r^{2m+1}}.$$ (A4)

The sum of exponents on x, y, and z is m−p. The total exponents on h is 2p+m−p+3−2, where 2p comes from ${\left(x^{\prime 2}+y^{\prime 2}+z^{\prime 2}\right)}^{p}$, m−p comes from ${\left(x^{\prime }x+y^{\prime }y+z^{\prime }z\right)}^{m-p}$, 3 from $\int_{-h/2}^{h/2}\int_{-h/2}^{h/2}\int_{-h/2}^{h/2}\text{d}x^{\prime }\text{d}y^{\prime }\text{d}z^{\prime }$, and −2 from $\frac{\partial }{\partial x_{i}^{\prime }}\frac{\partial }{\partial x_{j}^{\prime }}$. By defining n=m+p, we rewrite Eq. (A4) as

$$\sum_{n=0}\sum_{\;p=0}^{2p\leq n}d_{n-p,p,s,t}\frac{h^{n+1}x^{n-2p-s-t}y^{s}z^{t}{(x^{2}+y^{2}+z^{2})}^{\;p}}{r^{2n+1}}.$$ (A5)

For terms with odd n, before integration, the total exponents on $x^{\prime }$, $y^{\prime }$, and $z^{\prime }$ are n, thus those terms must be zero due to parity symmetry. The term with n=0 is also zero because, for n=0, m must also be zero, making it obvious. Therefore, only terms with n=2,4,6,… remain. After summing over p in Eq. (A5), we obtain Eq. (24).

Now turn to the *UMC* method. By replacing $r^{\prime }$ with $r^{\prime }-r^{\prime \prime }$ in Eq. (A1), and substituting it into Eq. (16), the demagnetization tensor element ${\left(N_{\text{c}}(r)\right)}_{ij}$ can be expressed in Cartesian coordinates as

$$\begin{array}{rl} & -\frac{1}{4\pi h^{3}}\sum_{m=0}a_{m}\int_{-h/2}^{h/2}\int_{-h/2}^{h/2}\int_{-h/2}^{h/2}dx^{\prime \prime }dy^{\prime \prime }dz^{\prime \prime }\\ & \;\times \int_{-h/2}^{h/2}\int_{-h/2}^{h/2}\int_{-h/2}^{h/2}dx^{\prime }dy^{\prime }dz^{\prime }\left(\frac{\partial }{\partial x_{i}^{\prime }}\frac{\partial }{\partial x_{j}^{\prime }}\right)\\ & \;\times ((x^{\prime }-x^{\prime \prime })x+(y^{\prime }-y^{\prime \prime })y+(z^{\prime }-z^{\prime \prime })z\\ & \;+{\left(x^{\prime }-x^{\prime \prime }\right)}^{2}+{\left(y^{\prime }-y^{\prime \prime }\right)}^{2}+{\left(z^{\prime }-z^{\prime \prime }\right)}^{2}){)}^{m}\frac{1}{r^{2m+1}}. \end{array}$$ (A6)

In the following, using the same strategy for the *UMCD* method, there is no major difficulty in obtaining Eq. (24) for the *UMC* method, although the process is more complex and the specific coefficients are different. We omit the detailed process here.

## APPENDIX B: SOLUTION FOR PROBLEM I

$H_{z}$ at r=0 for problem I is given by

$$\begin{array}{rl} & \frac{M_{0}}{12\pi {\left(\alpha +1\right)}^{2}(\alpha +2)}[-4\pi \alpha^{2}-18\alpha^{2}\log(2)-24\alpha^{2}\log\left(2-\sqrt{3}\right)+36\alpha^{2}\log\left(\sqrt{3}-1\right)-9\alpha^{2}\psi^{\left(0\right)}\left(\frac{\alpha +1}{4}\right)\\ & \;+9\alpha^{2}\psi^{\left(0\right)}\left(\frac{\alpha +3}{4}\right)-8\pi \alpha -36\alpha -54\alpha \log(2)-72\alpha \log\left(2-\sqrt{3}\right)+108\alpha \log\left(\sqrt{3}-1\right)\\ & \;-27\alpha \psi^{\left(0\right)}\left(\frac{\alpha +1}{4}\right)+27\alpha \psi^{\left(0\right)}\left(\frac{\alpha +3}{4}\right)-18\psi^{\left(0\right)}\left(\frac{\alpha +1}{4}\right)+18\psi^{\left(0\right)}\left(\frac{\alpha +3}{4}\right)\\ & \;-24\sqrt{2}\alpha^{2}F_{1}\left(\frac{\alpha +1}{2};-\frac{1}{2},1;\frac{\alpha +3}{2};-\frac{1}{2},-1\right)-36\sqrt{2}\alpha F_{1}\left(\frac{\alpha +1}{2};-\frac{1}{2},1;\frac{\alpha +3}{2};-\frac{1}{2},-1\right)\\ & \;-24\sqrt{2}F_{1}\left(\frac{\alpha +1}{2};-\frac{1}{2},1;\frac{\alpha +3}{2};-\frac{1}{2},-1\right)+18\sqrt{2}\alpha_{\;2}^{2}F_{1}\left(\frac{1}{2},\frac{\alpha +1}{2};\frac{\alpha +3}{2};-\frac{1}{2}\right)\\ & \;+18\sqrt{2}\alpha_{\;2}F_{1}\left(\frac{1}{2},\frac{\alpha +1}{2};\frac{\alpha +3}{2};-\frac{1}{2}\right)+12{\sqrt{2}}_{\;2}F_{1}\left(\frac{1}{2},\frac{\alpha +1}{2};\frac{\alpha +3}{2};-\frac{1}{2}\right)\\ & \;+18\alpha^{2}\Phi \left(-1,1,\frac{\alpha +3}{2}\right)+54\alpha \Phi \left(-1,1,\frac{\alpha +3}{2}\right)+36\Phi \left(-1,1,\frac{\alpha +3}{2}\right)-4\pi -72-36\log(2)\\ & \;-48\log\left(2-\sqrt{3}\right)+72\log\left(\sqrt{3}-1\right)], \end{array}$$ (B1)

where log is the natural logarithm, $\psi^{\left(0\right)}$ is the polygamma function of order 0, $F_{1}$ is the Appell series, F12 the hypergeometric function, and Φ is the Lerch transcendent.
